# ASPIRE: A multi-site community-based participatory research project to increase understanding of the dynamics of violence against immigrant and refugee women in Australia

**DOI:** 10.1186/s12889-015-2634-0

**Published:** 2015-12-23

**Authors:** Cathy Vaughan, Adele Murdolo, Linda Murray, Erin Davis, Jasmin Chen, Karen Block, Regina Quiazon, Deb Warr

**Affiliations:** Centre for Health Equity, Melbourne School of Population and Global Health, The University of Melbourne, Melbourne, VIC 3010 Australia; Multicultural Centre for Women’s Health, Suite 207, Level 2, 134 Cambridge St, Collingwood, VIC 3066 Australia; School of Medicine, Faculty of Health, University of Tasmania, Hobart, Tasmania, 7005 Australia

**Keywords:** Family violence, Immigrant, Refugee, Community-based research, Participatory research, Qualitative methods, Intersectionality, Australia

## Abstract

**Background:**

One in three women around the world are or have been subjected to violence. This includes in Australia, where violence against women is an urgent public health and human rights issue. Immigrant and refugee women who have resettled in Australia are known to face barriers accessing services aimed at preventing and responding to family violence. However there is little evidence about the contexts, nature and dynamics of violence against immigrant and refugee women to inform appropriate responses to enhance their safety and well-being. The ASPIRE project will address this gap by identifying opportunities for the development of responsive local and community-based interventions for family violence against immigrant and refugee women, contributing to the currently limited Australian research in this area.

**Methods/Design:**

This participatory research project will work with communities in eight geographic locations (two inner-city, three outer-suburban, and three regional) across two states (Victoria and Tasmania), to generate evidence about immigrant and refugee women’s experiences in a range of settings. The project will engage stakeholders and communities through extensive consultation prior to data collection and by facilitating community members’ participation in generating and analysing data. A mix of qualitative methods will be used to generate rich data about the family, cultural and place-based contexts that shape the prevalence and dynamics of violence against immigrant and refugee women; women’s prevention and help-seeking efforts; and community attitudes about and responses to violence across a range of cultural groups. Methods include in-depth interviews with women who have experienced family violence, key informant interviews with local community service providers, focus group discussions with men and women from predominant cultural groups that have migrated to areas covered by the research sites, and Photovoice with community leaders. Bilingual health educators will contribute to development of the research approach, the collection and analysis of data, and the dissemination of findings.

**Discussion:**

Findings from this two-year study will be disseminated to communities, service providers and policy-makers, providing evidence to inform culturally-appropriate prevention and support interventions, and building local communities’ awareness and capacity to respond to violence against immigrant and refugee women.

## Background

Violence against women is a public health, human rights and social policy problem that occurs in all communities and cultures. The prevalence of violence against immigrant and refugee women in Australia is unknown, with non-English speaking women under-represented in available quantitative data sets such as the Australian Personal Safety Survey. Surveys also underestimate violence against women with culturally and linguistically diverse backgrounds because of women’s varied perceptions of what constitutes violence, reluctance to discuss sensitive issues with unknown interviewers, and non-reporting of sexual violence in particular [1–3]. However it is known that a large proportion of people who have resettled in Australia since the 1980s have come from the three WHO regions with the highest lifetime prevalence of intimate partner violence and/or non-partner sexual violence – Africa, South and South-East Asia, and the Eastern Mediterranean [4, 5].

While prevalence data is incomplete, there is no evidence at this time that women who have resettled in Australia as immigrants or refugees experience higher rates of family violence than other Australian women. However the experiences associated with migration and resettlement are thought to increase the complexity of family dynamics and complicate the provision of effective support [6–8]. Available evidence suggests women who have resettled in Australia face barriers to services following family violence, including language barriers, logistical barriers, limited awareness of legal rights and of services, fear of police or that families will be broken up, social isolation, and shame [8–11]. Locally available services may be inadequately responsive to immigrant and refugee women’s needs [12, 13].

Literature on the dynamics of violence against immigrant and refugee women in Australia is limited. Research has highlighted how changing gender norms post-resettlement may increase women’s vulnerability to violence [7, 8, 14, 15]. Relationships between particular cultural values, violence-supportive attitudes, and violence against women have been analysed in some individual cultural communities [8, 9, 16]. Other factors shaping immigrant and refugee women’s experiences of violence, help-seeking, and access to services are less understood. In particular, little attention has been paid to the role of place – that is, to examining differences and similarities in the experiences of women who have resettled in Australian inner-city, outer-metropolitan, or regional settings; the local resources available to them; and the capacities of local communities to respond to violence.

Research elsewhere has highlighted the violence-protective effects of supportive neighbourhoods, and that local circumstances of socioeconomic disadvantage can potentially amplify risks of violence for women [17, 18]. Community members’ perceptions about their neighbourhood are known to impact upon women’s help-seeking behaviour [19], as does actual availability of responsive local services. However little evidence is available on Australian contexts. This project aims to address this gap by developing an in-depth understanding of the intersection of women’s migration experiences, place of resettlement, and experiences of violence and help-seeking in different settings. Findings will describe opportunities for supporting community-led responses to violence, informing violence prevention and support interventions and policy.

### Aims of ASPIRE

The project’s overall aim is to increase understanding of the nature and dynamics of violence against immigrant and refugee women in different Australian contexts. Specific research questions include:

1. What are immigrant and refugee women’s experiences of family violence and of help-seeking, in selected geographic communities in Australia?
2. What are local barriers and facilitators to immigrants and refugees accessing violence prevention and support services in different settings?
3. What opportunities exist for supporting community-led responses to family violence against immigrant and refugee women?

Data collection tools and processes are described below, and will assess relationships between women’s experiences of violence, help-seeking, access to services and:

- Current place of residence
- Visa type
- Length of time in Australia
- Membership of newly-arrived or established communities
- Access to networks of social support
- Features of the local environment (e.g. available services)
- Current relationship and family status
- Age (and age difference between women and their partners)
- Visible minority status
- Experiences of pre-arrival violence
- Knowledge of relevant laws
- Specific types of violence experienced by immigrant and refugee women, such as forced and early marriage, ‘honour killing’, multi-perpetrator domestic violence, and immigration status being used as a means of coercive control
- Other factors known to affect women’s vulnerability to violence (including women’s assets, disability, pregnancy; and women’s and men’s socio-economic status, employment and education).

This project will use the term ‘family violence’ consistent with the Victorian *Family Violence Protection Act 2008,* which defines family violence as behaviour by a person towards a family member of that person that is physically, sexually, economically, emotionally or psychologically abusive; threatening, coercive or controlling in such a way as to cause that family member fear. Behaviour by a person that causes a child to hear or witness or otherwise be exposed to the effects of these behaviors is also defined as family violence. This definition is inclusive of intimate partner violence (IPV), which is violence perpetrated by a current or former partner and is the focus of the Tasmanian *Family Violence Act 2004*. In addition, this project recognizes that family violence and IPV is most often and most severely perpetrated by men against their female partners and their children [20].

### Theoretical framework

Informed by feminist theory and practice, ongoing efforts to understand varied phenomena of violence against women have focused on the significance of gender and gender inequality in patriarchal social structures. However it is evident that gender inequality alone is insufficient for explaining how women experience violence across different socioeconomic, ethno-cultural and other circumstances. The concept of intersectionality and interlocking systems of oppression, first defined by Kimberlé Crenshaw in 1989 [21], is used to understand how immigrant and refugee women’s experiences of family violence are situated at a confluence of circumstances linked to social constructions of race, gender, sexuality, ethnicity and class, as well as policy and legal contexts, including immigration status and citizenship rights. Intersectional approaches enable us to consider how women’s experiences of family violence will differ across varied configurations of circumstances [22], as well as accounting for culturally specific contexts that render women vulnerable to experiencing family violence. Intersectional analyses allow for complex and nuanced insights into cultural differences, as well as consideration of how women’s circumstances are shaped by social, economic and political processes, minimising the risk of essentialising some cultures as more or less violent than others.

Critically, in order to address immigrant women’s ‘intersectional’ disadvantage “there is a need to engage our general public culture and institutions that perpetuate social inequalities and power differences” [23]. Researchers have proposed an approach that considers the *interactions* between the various sets of circumstances—emigration, reception in the migration country, socioeconomic circumstances, racial hierarchies and cultural heritage—as they bear on individuals’ experiences of migration and resettlement. There are a number of studies that have used intersectionality to gain insights into family violence experienced by immigrant and refugee women and avoid construing the ‘immigrant experience’ as homogenous [24]. Studies drawing on the concept of intersectionality show how related dynamics of displacement and marginalisation in immigrant communities are relevant for understanding structural and cultural contexts of family violence [25].

While intersectionality conceptualises the ways in which individuals’ understanding of social worlds are comprised of multiple subject positions, social ecological models position individuals within radiating sets of social determinants that influence potential for individual agency. These models have been developed to understand the social determinants of health and have been adapted to conceptualise social, economic and cultural determinants of family violence and violence against women. Lori Heise [26] first proposed an ecological framework for family violence that characterised gender-based violence as a result of multiple interactions that occur across various spheres of social life. This social ecological model has been instrumental in developing a model for understanding violence prevention and translating evidence generated through research into practical strategies [27]. Researchers have combined a social ecological model with theories of intersectionality to map the complex personal and social logics that are relevant for understanding the sociocultural patterning of family violence [28]. We have drawn on intersectional feminist theory and a social ecological model of family violence in the design of the project, and this theoretical framework will underpin analysis of our data.

## Methods/Design

ASPIRE is a community-based, qualitative research project that will be conducted across eight sites in two Australian states over a two-year period. The project involves generation and analysis of data drawn from in-depth interviews with immigrant and refugee women who have experienced family violence, key informant interviews with service providers working with immigrant and refugee women who have experienced family violence, focus group discussions with members of different cultural communities, and a Photovoice project with community leaders. Findings will increase understanding of the nature and dynamics of violence against immigrant and refugee women in diverse geographical locations.

The research team includes co-investigators from the University of Melbourne, Multicultural Centre for Women’s Health (MCWH), and University of Tasmania, and has recent experience working in partnership to conduct community-based research with immigrant and refugee women [29, 30]. The model of community engagement and participation developed through these projects informs the methodology outlined below. Immigrant and refugee women will be involved in all stages of the research including in the project’s initial design, in the development of research tools and processes, the collection and analysis of data, and in the presentation and dissemination of findings.

### Research sites

The research will be conducted in eight geographic communities in Victoria and Tasmania, including inner city, outer metropolitan and regional sites. Collectively the research team has strong relationships with communities and/or service providers in all proposed locations. The five Victorian sites include inner northwest Melbourne; City of Greater Dandenong; Brimbank City; Latrobe City; and the City of Greater Bendigo. The three sites in Tasmania include inner Hobart; Glenorchy City; and the City of Launceston.

These sites include communities with large and/or rapidly growing immigrant and refugee populations, including sites that have seen significant increases in newly-arrived immigrant and refugee groups in the last ten years (e.g. Launceston); sites with long-established immigrant communities (e.g. inner Melbourne); and sites with both established and newly-arrived immigrant and refugee communities (e.g. Brimbank City). Sites include a mix of communities well served by immigrant-specific services, as well as communities less service-rich. These sites will also enable the research team to engage with participants from a range of cultural communities. For example in the City of Greater Dandenong (the most culturally diverse local government area in Victoria), we will recruit participants from established communities (such as the Vietnamese, Indian, Sri Lankan, and Cambodian communities), as well participants from more recently arrived and rapidly growing cultural communities (such as the Afghan, Pakistani, Burmese and Sudanese communities).

### Initial consultations

Our experience suggests a pre-data collection consultative phase is vital to a community-based and participatory approach, enabling community input into research questions and methods, and ensuring genuine engagement and participation in future activities.

The research team has held consultations with state-wide and local stakeholders in Victoria and Tasmania, including representatives from violence prevention and response and the immigrant and refugee services sector. These consultations have informed the research team about stakeholder priorities for research on family violence with immigrant and refugee communities; relevant cultural, immigrant and refugee communities in each location; stakeholders’ past experience of research on family violence in their local area, both positive and negative; perspectives about proposed research approaches and tools; and availability of local services, support mechanisms and referral pathways for women participating in research activities.

The research team has also consulted with bilingual-bicultural health educators (BHEs) employed by the Multicultural Centre for Women’s Health who represent cultural communities prominent in the research sites. The BHEs provided feedback on their experiences conducting research in their communities; their experience of working in response to violence in their communities; potential barriers to community members’ participation and how these barriers may best be overcome; approaches to community engagement and recruitment strategies; and our proposed research methods and initial drafts of research tools.

### Training and capacity building

The research design includes a substantive investment in BHE training to ensure that all members of the research team have the skills and confidence to be actively and safely involved throughout the project. Training will aim to ensure a shared understanding of the aims and objectives of the project; family violence; the ethical considerations associated with the project; and the project safety protocol. Workshops will also aim to increase co-investigator understanding of the different local contexts, of BHE experience and expertise, and the sensitivities and considerations that there may be in conducting research on family violence with specific cultural communities. Training workshops will be conducted in Melbourne, Hobart and Launceston. The co-investigators will work closely with the large team of BHEs throughout the project, debriefing after data collection activities, jointly translating and transcribing audio-recordings, and during initial participatory analysis of data. This will enable co-investigators to play an ongoing mentoring role to build the research capacity of BHEs, and for BHEs to provide regular and timely feedback about the conduct of research activities.

### Data collection

In each of the Victorian and Tasmanian sites the research team will use recruitment methods as advised by stakeholder consultations. We will collect data using a combination of key informant interviews (with service providers), focus group discussions (with up to two groups of women and one group of men from different cultural communities at each site), and in-depth interviews (with immigrant and refugee women who have experienced family violence), to generate and triangulate rich qualitative data. All interviews and focus group discussions will be audio-recorded with participant permission. Participants in ASPIRE may be on temporary visas or have permanent residence. They may have come to Australia through marriage migration sponsored by Australian residents or citizens; be international students studying or living in the research sites; be seeking asylum in Australia; be on one of the many temporary visas available, including temporary work visas; or have come to the country permanently through the skilled migration, humanitarian or family streams of the migration program.

### Key informant interviews

At each of the research sites we will conduct key informant interviews with three to five local service providers (a minimum of 24 interviews in total). Depending on the site, key informants may include representatives from domestic violence services, health services, settlement and ethno-specific services, and/or the law and justice sector. Interviews will be conducted in English by an academic member of the research team, be up to 60 min long, and will explore service providers’ perceptions of local service needs, area-level characteristics, barriers and facilitators to community members accessing services, and current community-led responses.

Key informants will be recruited through Family Violence Regional Integration Committees (in Victoria); researchers’ and project Advisory Group members’ networks; and emails sent to local service providers and relevant organisations.

### Focus group discussions

At each research site we will conduct focus group discussions (FGDs) with two groups of women and one group of men from different cultural communities prominent in that particular project site (a minimum of 24 FGDs in total). During the FGDs we will not ask about participants’ experiences of violence but will explore community perceptions and values related to gender, the impact of migration and re-settlement on family life, access to local services, and violence prevention and intervention in general. FGDs will take approximately 90 min and be facilitated by a BHE (in the relevant language for participants) or an academic member of the research team where English is appropriate. All FGDs will be single sex groups, with discussions held with groups of women and groups of men separately. During FGDs conducted in a language other than English, an interpreter will be contracted to provide simultaneous interpretation to an academic member of the research team who will be the note-taker for the group.

Participants for FGDs will be recruited through community associations, researcher and BHE networks, local immigrant and refugee services, and groups that already meet in relation to family violence (e.g. culturally specific women’s support groups).

### In-depth interviews with women who have experienced family violence

At each of the research sites, in-depth interviews will be conducted with six to eight immigrant or refugee women, or women seeking asylum in Australia, who have experienced family violence. Approximately 48 interviews will be conducted. Recruitment of participants will continue until theoretical saturation is achieved within the data. Question guides will be designed to explore women’s experiences of violence, help-seeking, services, and local supports. Interviewers will also complete a short demographic questionnaire with the interviewees to collect information about their age, country of birth, language spoken at home, length of time in Australia, visa status, education and employment; similar information will also be collected about the primary person using violence against the interviewees. Interviews will take approximately 90 min and will be conducted in the participant’s preferred language and by their preferred interviewer option (i.e. an academic researcher in English, with or without an interpreter, or by a bilingual-bicultural health educator).

Potential participants for interview will be identified through local domestic violence and resettlement/multicultural services, as well as the networks of the trained bilingual-bicultural health educators. Members of the research team will visit cultural community groups in the research sites to disseminate information about the project. During these visits a member of the research team will provide project information flyers, answer questions and invite women interested in participating in interviews to contact the research team. Information about the project will also be disseminated through multi-language programs on radio and through service providers. Participants in focus group discussions may also later participate in in-depth interviews if they would like to share their own experiences in relation to family violence.

### Photovoice

Female and male leaders from participating communities will also be invited to participate in a Photovoice project. Photovoice is a participatory research method that enables participants to share their experiences, perspectives and priorities through photography [31]. Visual research methods can generate rich insights into sensitive topics difficult to express in words, and generate compelling data for translation into policy, practice and community-led social change [24, 32-34]. Photovoice, specifically, facilitates safe social spaces for disadvantaged groups to creatively communicate about difficult issues [35–37], including violence [38–40] and resettlement [41]. Interested community leaders at each site will be provided with intensive training in the method (with particular focus on ethical considerations), and supported to generate photo-stories communicating their perspectives on the need for, approaches to, and opportunities to support, community-led responses to family violence.

Potential Photovoice participants will be identified in consultation with women from the participating cultural communities and the BHEs, as individuals recognised as community leaders may not always be the most appropriate people to lead community responses on the specific issue of family violence.

### Data analysis

All interviews and discussions will be digitally audio-recorded with participant permission; in addition notes will be taken by the interviewer or by the note-taker in FGDs. Audio-recordings will be transcribed and translated (where necessary). Where permission is not obtained for audio-recording, or sound quality precludes accurate transcription (as is sometimes the case with recordings from FGDs) researchers’ detailed notes will be written up for inclusion in the analysis; whether data are derived from notes or a transcript will be noted during analysis.

Initial review of the transcripts will use a deductive process, based on concepts drawn from the literature on violence against immigrant and refugee women. In particular, an intersectional feminist theoretical framework will inform early analysis of the data. This early deductive analysis of data will be done collaboratively with the BHEs during a participatory data analysis workshop. This workshop will enable the BHEs to contribute to data analysis and give cross-cultural insight into emerging themes, and will lead to the development of a draft data coding framework. This coding framework will then be refined based on an inductive ‘data-driven’ process with codes identified from the empirical material. Coding of data will occur across the entire dataset, which will help to identify themes [42]. Given the size of the dataset, NVivo software will be used to manage the process and to categorise data into themes. Data coding will be completed by two members of the research team, with the Principal Investigator also using the coding framework to code a selection of data, to assess for inter-coder reliability.

All FGD participants, community stakeholders and service providers will be invited to a community feedback workshop at each site to be held after data collection is complete and data have been subject to early analysis. An overview of early findings will be presented to participating communities to give them the opportunity to reflect upon and interrogate researchers’ interpretation and analysis of the data. All data presented in these workshops will be de-identified and any potentially identifying details excluded. These workshops will enable community input into the final project report and publications emerging from the project.

### Participant and researcher safety

Women experiencing family violence are already exposed to numerous risks to their physical, emotional/psychological, social, and economic well-being, which may in turn have an impact on their safe participation in research [43]. A comprehensive Safety Protocol has been developed to provide guidance on managing risks associated with all aspects of the ASPIRE project, including risks for participants and for members of the research team.

The Safety Protocol draws on existing ethical and safety guidelines for researching women’s experiences of violence [44–50]. In addition, focus group discussions involving men will be informed by guidelines for investigating men’s perceptions of gender-based violence [51, 52], though it should be noted that these focus group discussions should not be considered research with perpetrators but rather will examine group norms and attitudes.

The research team recognise that family violence can produce trauma and considerable mental health impacts with consequences such as depression, anxiety or suicide [53]. Further, immigrants and particularly refugees may also carry lingering mental health effects from exposure to traumatic events in their countries of origin or in camps and detention centres prior to settlement [54] and may be vulnerable to experiencing intense emotions through the research process. FGD and in-depth interview questions have been carefully designed to minimise the potential risk of participants becoming distressed and training has been provided to support all members of the research team to take a trauma-informed approach.

### Other ethical considerations

The study will be conducted in accordance with the tenets of the Declaration of Helsinki. All participants will be informed about the study using a plain language statement. Plain language statements for the project will be available in English and will be translated for a number of the key language groups. Where participants are not able to read the plain language statement, it will be read to them in a language that they understand. All interested participants will be asked to provide written informed consent to participate and for publication of findings, noting that they will not be individually identified. All participants will be aged 18 years or over and have the ability to provide independent voluntary consent. Participants who are assessed by service providers and/or the research team as being in the middle of a current crisis or any situation that could be aggravated by involvement with the research will be excluded for safety reasons. The University of Melbourne Human Research Ethics Committee (ethics ID 1544857.1) and the Tasmania Social Sciences Human Research Ethics Committee (ethics ID H0015235) have granted ASPIRE ethics approval.

## Discussion

Twenty-eight percent of the Australian population was born overseas [55], and yet the needs of immigrant and refugee women experiencing family violence are inadequately addressed in existing national responses, policies and services. In part this is due to the very limited availability of information available to inform appropriate and effective responses. ASPIRE will provide policy-makers, service providers and communities with an in-depth analysis of the nature of violence against immigrant and refugee women and women’s experiences of this violence, across and within particular communities in diverse Australian settings. Knowledge generated will have a particular focus on the intersections between place, migration, gender and culture. Information generated will provide evidence to inform culturally appropriate prevention and support interventions, and can contribute to building local communities’ awareness and capacity to respond to violence against immigrant and refugee women.

Research exploring violence against immigrant and refugee women in Australia has tended to be small scale and focus on one specific cultural community in one particular place. Those few studies that have examined the experience of more than one cultural group have, however, been limited to understanding the experience of people who came to Australia as refugees [7, 14, 56]. This project will make a unique contribution to the literature, examining commonalities and differences in the experience of refugees, asylum seekers and migrants. We will be able to explore the impact of visa status on women’s experiences and on service providers’ ability to meet women’s needs. ASPIRE will also contribute to the very limited understanding of how place of resettlement, that is whether women and families resettle in inner-city, outer metropolitan, or regional areas, affects their experience of family violence and the help that they can access.

Few study protocols for multi-site community-based participatory research projects have been published. We hope that by outlining the processes used in the ASPIRE project we can encourage engagement with bilingual-bicultural workers as invaluable resources in cross-cultural research. We anticipate that the approach used in ASPIRE will be of interest to others analysing the complex intersections of place, gender and culture as experienced by immigrant and refugee women, and as they shape the dynamics of family violence in diverse communities.

## Abbreviations

ASPIRE: Analysing safety and place in immigrant and refugee experience
BHE: Bilingual health educators
FGD: Focus group discussion
IPV: Intimate partner violence
MCWH: Multicultural Centre for Women’s Health

## References

[1] Lievore D. Non-reporting and hidden recording of sexual assault: An international literature review. Commonwealth Office of the Status of Women. 2003. http://www.aic.gov.au/media_library/archive/publications-2000s/non-reporting-and-hidden-recording-of-sexual-assault-an-international-literature-review.pdf. Accessed 22 June 2015.

[2] Mouzos J, Makkai T. Women’s experiences of male violence: Findings from the Australian Component of the International Violence Against Women Survey (IVAWS). Australian Institute of Criminology. 2004. http://www.aic.gov.au/media_library/publications/rpp/56/rpp056.pdf. Accessed 22 June 2015.

[3] Ghafournia N (2011). Battered at home, played down in policy: Migrant women and domestic violence in Australia. Aggress Violent Behav..

[4] Devries K, Mak J, Garcia-Moreno C, Petzold M, Child J, Falder G (2013). The global prevalence of intimate partner violence against women. Sci.

[5] World Health Organization. Global and regional estimates of violence against women: Prevalence and health effects of intimate partner violence and non-partner sexual violence. WHO. 2013. http://apps.who.int/iris/bitstream/10665/85239/1/9789241564625_eng.pdf?ua=1. Accessed 1 June 2015.

[6] Bartolomei L, Eckert R, Pittaway E (2014). "What happens there… follows us here": Resettled but still at risk: refugee women and girls in Australia. Refuge.

[7] Fisher C (2013). Changed and changing gender and family roles and domestic violence in African refugee background communities post-settlement in Perth Australia. Violence Against Women.

[8] Zannettino L (2012). “… There is no war here; it is only the relationship that makes us scared”: Factors having an impact on domestic violence in Liberian refugee communities in South Australia. Violence Against Women.

[9] Taft A, Small R, Hoang K (2008). Intimate partner violence in Vietnam and among Vietnamese diaspora communities in western societies: A comprehensive review. J Fam Stud..

[10] Drummond P, Mizan A, Brocx K, Wright B (2011). Barriers for accessing health care services for West African refugee women living in Western Australia. Health Care Women Int..

[11] Vatcharavongvan P, Hepworth J, Lim J, Marley J (2014). What are the health needs, familial and social problems of Thai migrants in a local community in Australia? A focus group study. J Immigr Minor Health.

[12] Poljski C. On Her Way: Primary prevention of violence against immigrant and refugee women in Australia. Multicultural Centre for Women’s Health. 2011. http://www.mcwh.com.au/downloads/publications/On_Her_Way_2011.pdf. Accessed 1 June 2015.

[13] Murdolo A (2014). Safe homes for immigrant and refugee women: Narrating alternative histories of the women’s refuge movement in Australia. Front.

[14] Rees S, Pease B (2007). Domestic violence in refugee families in Australia. J Immigr Ref Stud..

[15] James K (2010). Domestic violence within refugee families: Intersecting patriarchal culture and the refugee experience. Aust N Z J Fam Ther.

[16] Taylor N, Mouzos J. Community Attitudes to Violence Against Women Survey 2006: A full technical report. Australian Institute of Criminology. 2006. http://www.aic.gov.au/media_library/archive/publications-2000s/community-attitudes-to-violence-against-women-survey-a-full-technical-report.pdf. Accessed 1 June 2015.

[17] Daoud N, O’Campo P, Urquia M, Heaman L (2012). Neighbourhood context and abuse among immigrant and non-immigrant women in Canada: findings from the maternity experiences survey. Int J Public Health.

[18] Frye V, O’Campo P (2011). Neighborhood effects and intimate partner and sexual violence: Latest results. J Urban Health.

[19] McDonnell K, Burke J, Gielen A, O’Campo P, Weidl M (2011). Women’s perceptions of their community’s social norms towards assisting women who have experienced intimate partner violence. J Urban Health.

[20] Heise L. What works to prevent partner violence? An evidence overview. STRIVE. 2011. http://strive.lshtm.ac.uk/resources/what-works-prevent-partner-violence-evidence-overview. Accessed 1 June 2015.

[21] Crenshaw K (1989). Demarginalizing the Intersection of Race and Sex: A Black Feminist Critique of Antidiscrimination Doctrine, Feminist Theory and Antiracist Politics. Univ Chic Leg Forum.

[22] Sokoloff NJ, Dupont I (2005). Domestic violence at the intersections of race, class, and gender: challenges and contribution to understanding violence against marginalized women in diverse communities. Violence Against Women.

[23] Pearce SC, Sokoloff NJ (2013). "This should not be happening in this country": private-life violence and immigration intersections in a U.S. Gateway City. Sociol Forum.

[24] Alaggia R, Maiter S, Ramona Alaggia R, Vine C (2006). Domestic violence and child abuse: Issues for immigrant and refugee families. Cruel but Not Unusual: Violence in Canadian Families.

[25] Sokoloff NJ (2008). Expanding the intersectional paradigm to better understand domestic violence in immigrant communities. Critical Crim..

[26] Heise L (1998). Violence against women: an integrated, ecological framework. Violence Against Women.

[27] Michau L, Horn J, Bank A, Dutt M, Zimmerman C (2015). Prevention of violence against women and girls: lessons from practice. Violence against women and girls 4. Lancet.

[28] Guruge S, Khanlou N (2004). Intersectionalities of influence: researching the health of immigrant and refugee women. Can J Nurs Res..

[29] Vaughan C, White N, Keogh L, Tobin J, Ha B, Ibrahim M, et al. Listening to North Yarra Communities about female genital cutting. The University of Melbourne. 2014 http://www.socialequity.unimelb.edu.au/wp-content/uploads/2013/01/Listening-to-North-Yarra-Communities-WEB.pdf. Accessed 1 June 2015.

[30] Vaughan C, White N, Keogh L, Tobin J, Murdolo A, Quiazon R, et al. Female genital mutilation/cutting in regional Victoria: Research to practice. The University of Melbourne. 2014. http://www.netfa.com.au/downloads/FGMC-in-Regional-Victoria-Report-WEB.pdf. Accessed 1 June 2015.

[31] Wang C, Burris MA (1997). Photovoice: Concept, methodology, and use for participatory needs assessment. Health Educ Behav.

[32] Rees S, Pease B. Refugee Settlement, Safety and Wellbeing: Exploring domestic and family violence in refugee communities. VicHealth. 2006. http://library.bsl.org.au/jspui/bitstream/1/781/1/Refugee_settlement_safety_and_wellbeing.pdf. Accessed 22 June 2015.

[33] Cannuscio C, Weiss E, Fruchtman H, Schroeder J, Weiner J, Asch D (2009). Visual epidemiology: Photographs as tools for probing street-level etiologies. Soc Sci Med.

[34] Orzanne J, Moscato E, Kunkel D (2013). Transformative photography: evaluation and best practices for eliciting social and policy changes. J Public Policy Mark.

[35] Vaughan C (2010). ‘When the road is full of potholes, I wonder why they are bringing condoms?’ Social spaces for understanding young Papua New Guineans’ health-related knowledge and health-promoting action”. AIDS Care.

[36] Vaughan C (2014). Participatory research with youth: Idealising safe social spaces or building transformative links in difficult environments?. J Health Psychol.

[37] Vaughan C, Rohleder P, Lyons A (2014). Participatory research. Qualitative Research in Clinical and Health Psychology.

[38] Frohmann L (2005). The Framing Safety Project: Photographs and narratives by battered women. Violence Against Women.

[39] Ponic P, Jategaonkar N (2012). Balancing safety and action: Ethical protocols for photovoice research with women who have experienced violence. Arts Health.

[40] Chonody J, Ferman B, Amitrani-Welsh J, Martin T (2013). Violence through the eyes of youth: A photovoice exploration. J Community Psychol.

[41] Sutherland C, Cheng Y (2009). Participatory-action research with (im)migrant women in two small Canadian cities: Using Photovoice in Kingston and Peterborough Ontario. J Immigr Refug Stud..

[42] Braun V, Clarke V (2006). Using thematic analysis in psychology. Qual Res Psychol.

[43] Kyriakakis S, Waller B, Kagotho N, Edmong T (2014). Conducting safe research with at-risk populations: Design strategies from a study with unauthorized migrant women experiencing intimate abuse. Qual Soc Work.

[44] World Health Organization. Putting women first: Ethical and safety recommendations for research on domestic violence against women. WHO. 2001. http://www.who.int/gender/violence/womenfirtseng.pdf . Accessed 22 June 2015.

[45] Ellsberg M, Heise L (2002). Bearing witness: ethics in domestic violence research. Lancet.

[46] Fontes L (2004). Ethics in violence against women research: The sensitive, the dangerous and the overlooked. Ethics Behav.

[47] Sullivan C, Cain D (2004). Ethical and safety considerations when obtaining information from or about battered women for research purposes. J Interpers Violence.

[48] Ellsberg M, Heise L (2005). Researching Violence Against Women: A practical guide for researchers and activists.

[49] National Health and Medical Research Council. National Statement on Ethical Conduct in Human Research. NHMRC; 2007. http://www.nhmrc.gov.au/_files_nhmrc/file/publications/synopses/e72.pdf. Accessed 1 June 2015.

[50] World Health Organization. Responding to intimate partner violence and sexual violence against women: WHO clinical and policy guidelines. WHO; 2013. http://apps.who.int/iris/bitstream/10665/85240/1/9789241548595_eng.pdf. Accessed 1 June 2015. 24354041

[51] Jewkes R, Dartnall E, Sikweyiya Y. Ethical and safety recommendations for research on the perpetration of sexual violence. Sexual Violence Research Initiative; 2012. http://www.svri.org/EthicalRecommendations.pdf. Accessed 1 June 2015.

[52] Fulu E, Lang J. Ethical and safety guidelines for research on gender-based violence. Partners for Prevention; 2013. http://www.partners4prevention.org/sites/default/files/ethical_and_safety_guidelines_for_research_with_men_final.pdf. Accessed 1 June 2015.

[53] Dutton M, Green B, Kaltman S, Roesch D, Zeffiro T, Krause E (2006). Intimate partner violence, PTSD and adverse health outcomes. J Interpers Violence.

[54] Abraham M (2000). Isolation as a Form of Marital Violence: The South Asian Migrant Experience. J Soc Distress Homel.

[55] Australian Bureau of Statistics. Australia’s population by country of birth. ABC; 2015. http://www.abs.gov.au/ausstats/abs@.nsf/Lookup/3412.0Chapter12011-12%20and%202012-13. Accessed 22 June 2015.

[56] Pittaway E, Muli C, Shteir S (2009). I have a voice – hear me!” Findings of an Australian study examining the resettlement and integration experience of refugees and migrants from the Horn of Africa in Australia. Refuge.

